# Pervasive Local-Scale Tree-Soil Habitat Association in a Tropical Forest Community

**DOI:** 10.1371/journal.pone.0141488

**Published:** 2015-11-04

**Authors:** Elodie Allié, Raphaël Pélissier, Julien Engel, Pascal Petronelli, Vincent Freycon, Vincent Deblauwe, Laure Soucémarianadin, Jean Weigel, Christopher Baraloto

**Affiliations:** 1 Université des Antilles et de la Guyane, UMR EcoFoG, Kourou, France; 2 IRD, UMR AMAP, Montpellier, France; 3 CNRS, UMR EcoFoG, Kourou, France; 4 Cirad, UMR EcoFoG, Kourou, France; 5 Cirad, UPR BSEF, Montpellier, France; 6 UMR Diade, Montpellier, France; 7 Cirad, UMR EcoFoG, Kourou, France; 8 AgroParisTech, UMR EcoFoG, Kourou, France; 9 INRA, UMR EcoFoG, Kourou, France; 10 International Center for Tropical Botany, Department of Biological Sciences, Florida International University, Miami, FL, United States of America; CNRS / Université Joseph-Fourier, FRANCE

## Abstract

We examined tree-soil habitat associations in lowland forest communities at Paracou, French Guiana. We analyzed a large dataset assembling six permanent plots totaling 37.5 ha, in which extensive LIDAR-derived topographical data and soil chemical and physical data have been integrated with precise botanical determinations. Map of relative elevation from the nearest stream summarized both soil fertility and hydromorphic characteristics, with seasonally inundated bottomlands having higher soil phosphate content and base saturation, and plateaus having higher soil carbon, nitrogen and aluminum contents. We employed a statistical test of correlations between tree species density and environmental maps, by generating Monte Carlo simulations of random raster images that preserve autocorrelation of the original maps. Nearly three fourths of the 94 taxa with at least one stem per ha showed a significant correlation between tree density and relative elevation, revealing contrasted species-habitat associations in term of abundance, with seasonally inundated bottomlands (24.5% of species) and well-drained plateaus (48.9% of species). We also observed species preferences for environments with or without steep slopes (13.8% and 10.6%, respectively). We observed that closely-related species were frequently associated with different soil habitats in this region (70% of the 14 genera with congeneric species that have a significant association test) suggesting species-habitat associations have arisen multiple times in this tree community. We also tested if species with similar habitat preferences shared functional strategies. We found that seasonally inundated forest specialists tended to have smaller stature (maximum diameter) than species found on plateaus. Our results underline the importance of tree-soil habitat associations in structuring diverse communities at fine spatial scales and suggest that additional studies are needed to disentangle community assembly mechanisms related to dispersal limitation, biotic interactions and environmental filtering from species-habitat associations. Moreover, they provide a framework to generalize across tropical forest sites.

## Introduction

The assembly of tropical tree communities remains a subject of rich historical debate [1]. Notably, the importance of assembly processes may vary greatly between localities studied [2] and may interact at different spatial scales in a given locality [3–5]. The high beta and gamma diversity of tropical tree communities, with species pools in the hundreds to thousands at large spatial scales (> 100 ha), is perhaps not surprising given the high degree of environmental heterogeneity and frequency of geographic barriers at these landscape scales [2,6–8]. However, the large number of species that occur at small spatial scales suggests that habitat partioning at broad scale alone is insufficient to explain local tree species diversity [9,10]. More controversy exists regarding the extent to which processes such as environmental filtering structure local communities at small spatial scales (< 100 ha). Increasing evidence implicates biotic interactions exerting strong controls on local community structure in tropical forests [11–13]. However, other studies demonstrate the importance of small-scale environmental heterogeneity in shaping local community structure [10,14–17] by environmental filtering. Consensus is therefore lacking regarding the frequency with which tree species distributions are constrained to particular habitats, with evidence for species-habitat association of tropical trees ranging from 25−82% [18]. We submit that the existing discord may be due to differences in scale and in the methods used [18,19].

The diversity in methods employed to examine species-habitat associations in tropical tree communities has developed to respond to two major challenges. First is the characterization of habitats, ranging from simple classifications of broad habitat types [20,21] to definitions of complex multivariate gradients [14]. Several authors argued that it is necessary to integrate not only topography but also soil fertility gradients to capture fine scale associations within diverse tree communities [14,18,22,23]. A second challenge involves the decoupling of inherent autocorrelation in species and/or habitat spatial structure. Indeed, most ecological variables were spatially autocorrelated and the majority of tropical tree species have aggregated population structures at some spatial scale [15,24–27]. Aggregated patterns may result either from a functional dependence between species requirements and habitat characteristics, from inherent population dispersal dynamics or from inter-specific interactions, all processes being actually in action to determine the observed spatial patterns featuring the realized species niches. It follows that apparent species-habitat concordance may exist even when species and habitat are independently spatially structured, making most statistical tests of species-habitat associations that assume independence of observations too liberal [28]. Several methods have been proposed to overcome this bias and to appropriately define species-habitat associations. The most popular in ecology is probably the torus translation test [10], which rotates environmental maps while maintaining the spatial structure of tree populations. However, the torus translation test provides inflated type I error rates in case of highly correlated data because of the introduction of artificial linear frontiers when wrapping back the edge of the map onto the torus [29]. A novel method is instead proposed by [29], based on an image synthesis technique that preserves autocorrelation of original maps when building the probability distribution of the test statistics (Pearson's correlation coefficient when the two maps are raster images) thus providing an unbiased test of spatial association. However this new technique has never been applied for robustly testing systematic species-habitat associations in ecological communities.

A major challenge in tropical community ecology remains extrapolating from the tens or hundreds of focal species whose abundance permits analysis of habitat preferences [14,15,18], to the tens of thousands of species estimated to occur in most tropical regions [8,30]. We might extrapolate patterns of habitat preferences to understudied taxa using the less-intensive description of functional strategies of these taxa, which are hypothesized to correlate with species distributions [31–33]. For example, across broad environmental gradients, leaf and wood tissues tend to be more dense in habitats with limited soil nutrients and increased drought stress [34]. Across local topographic gradients that represent differences in light availability and forest turnover rate, [35] predict species composition dominated by light-wooded fast-growing species on seasonally inundated bottomlands and by dense-wooded slow-growing species on plateaus.

Here we integrate the novel correlation test based on image synthesis to assess species-habitat associations with a comprehensive dataset of replicated permanent plots, large enough (6.25 ha each) to study the local distribution of species across broad gradients of topography, soil hydrology and fertility. We analyze the distribution of more than 15,800 trees of 94 species in 37.5 ha of lowland forest to understand how environmental heterogeneity correlates with the spatial distribution of species at a local scale. In particular, we address the following questions:

1. Which environmental factors (topography, soil hydrology and soil fertility) contribute to fine-scale heterogeneity, and how??
2. How frequent is species-habitat association across these environmental gradients?
3. Which environmental factors filter tree species distributions, and how?
4. To what extent do environmental gradients filter for species with similar functional strategies?

We discuss how we can generalize from our results to a more comprehensive evaluation of how environmental heterogeneity shapes tropical tree community structure.

## Materials and Methods

Cirad (owner of the Paracou experimental station) gave permission to conduct the study on this site.

### Study site

**Fig 1:**
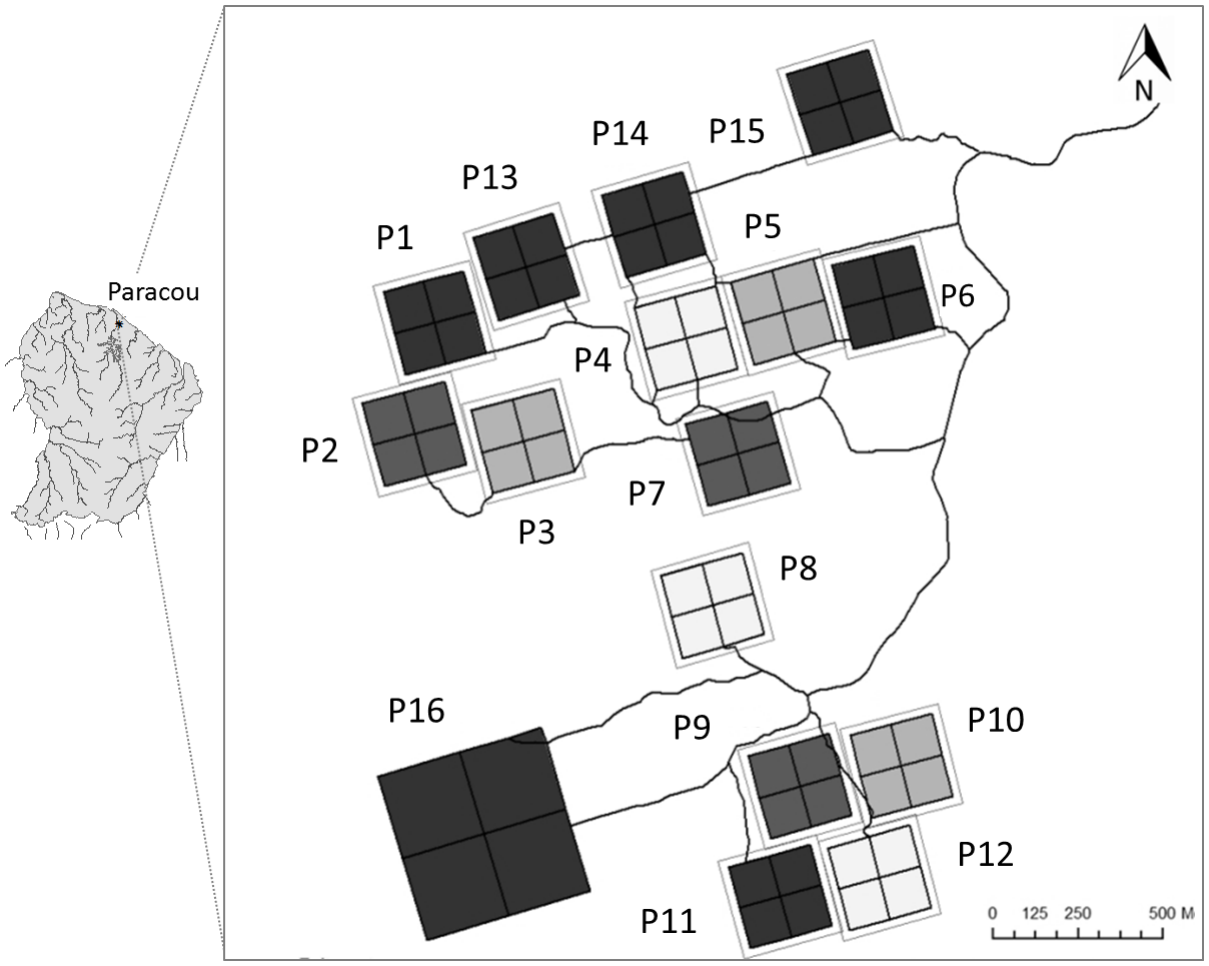
Study site. The study site is located at the experimental station of Paracou (5°18’N, 52°55’W), French Guiana, in a lowland tropical rainforest. Six 6.25-ha control plots (small black squares) spread over an area of ca. 50 km^2^ were considered in this study. Source of maps: SIG data of EcoFoG (Cirad Guyane).

The study site is located at the Paracou experimental station (5°18’N, 52°55’W), a lowland tropical rain forest in French Guiana (Fig 1). Nearly 60% of the 3160 mm annual precipitation falls during the period between mid-April and mid-June, and less than 50 mm per month in September and October [36]. This site is located on schist soils with veins of pegmatite and along a Precambrian metamorphic formation called the Bonidoro series [37], corresponding to acrisols in the WRB international soil classification [38]. Some topographic variation exists within this site, with absolute altitudes ranging from 0 to 41.7 m, and slope from 0 to 18 degrees relatives to the horizontal. Soil conditions are heterogeneous at Paracou, and several studies have indicated that many soil physical and chemical properties are correlated with topographic position [20,35,39]. The site contains high species diversity, with 160 species per hectare[40]. Six 6.25 ha permanent plots (totaling 37.5 ha) were established across ca. 50 km^2^ at the site (Fig 1) and have been inventoried bi-annually since the mid-1990s. Here we concentrate on the most recent inventory of these plots in 2013.

### Botanical data

All species with stem ≥ 10 cm in Diameter at Breast Height (DBH) have been identified for the 22,810 trees in the six studied plots, with vouchers determined at the regional herbarium (CAY) and by appropriate taxonomic specialists. A total of 508 distinct morpho-species was identified in this dataset. For this study, we selected species based on several criteria. First, we restricted our analysis to the more common species to avoid spurious species-habitat associations detected for rare taxa. We selected species based on (i) an overall density above one individual per hectare [41] and (ii) at least one individual in each of the six 6.25-ha plots. Second, we removed the two common palm species (*Oenocarpus bataua* and *O*. *bacaba*) from the analysis to restrict our comparison to the guild of dicots. The resulting dataset contained 94 species from 67 genera and 30 families (with dominant families Lecythidaceae, Chrysobalanaceae, Fabaceae, Sapotaceae), representing 15,823 individuals (S1 Table). Four of these taxa are morphospecies that are in the process of taxonomic description (*Chaetocarpus sp*. *1*, Euphorbiaceae; *Symphonia sp*. *1*, *Tovomita sp*. *2 (DS)*, *Tovomita sp*. *P4*, Clusiaceae).

### Environmental data

To test species-habitat associations we first created maps of environmental factors recognized to be representative of habitat heterogeneity and potentially important for the spatial structure of tree communities. In French Guiana, the local hydrological, physical and geochemical soil features resulting from the weathering process along topographic catenas have been recognized as predominant ecological factors acting on the tree community [20,35,39,42]. We thus derived continuous environmental maps related to topography, soil hydrology and fertility (Table 1 and S1 File and S2 File and S3 File) against which to contrast tree species distributions.

**Table 1:** Characteristics of environmental variables mapped to test species-habitat associations.

Environmental Variables	Abreviations	Soil characteristics	Description	Range	Units
**Asbolute elevation**	AbsEl	topography	Digital terrain model (DTM) derived from LIDAR (Light Detection And Ranging) data	0−42	meters
**Relative elevation**	RelEl	topography	Relative altitude above the nearest stream	0−17	meters
**Local slope angle**	Slope	topography	Estimation of slope angle deviation from the horizontal plane	0−18	degrees
**Wetness Index**	Wetness	soil hydrology	Estimation of soil moisture	1−12	NA
**Flow accumulation**	FlAc	soil hydrology	Estimation of surface runoff	0−1800	NA
**Total C content**	C	organic matter	Concentration of total C content in soil	0−28	g.kg^-1^
**Available P content**	P	soil fertility	Concentration of available P content in soil	0−6	mg.kg^-1^
**H exchangeable**	H	acidity	Concentration of H exchangeable in soil	0−0,8	cmolc.kg^-1^
**Al exchangeable**	Al	acidity and toxicity	Concentration of Al exchangeable in soil	0,4−1,7	cmolc.kg^-1^
**Base-cation saturation ratio**	BS	soil fertility	Saturation of base-cation on CEC	4−13	%

Topography was described with absolute elevation, relative elevation from the nearest stream and local slope angle. Absolute elevation was calculated as a digital terrain model (DTM) derived from LIDAR (Light Detection And Ranging) data acquired across the Paracou site in October 2009. Relative elevation from the nearest stream, local slope angle, flow accumulation and wetness index were calculated from characteristics of DTM using the package “Terrain Analysis” from SAGA GIS (System for Automated Geoscientific Analyses Geographic Information Systems) software [43]. Relative elevation represents relative altitude above the nearest stream, which is likely to be more pertinent to local hydrological characteristics than absolute altitude. Local slope angle measures estimated deviation in relation to the horizontal plane. Hydrology was estimated as flow accumulation and wetness index. Flow accumulation estimates surface runoff with the Mass-Flux Method [44]. Wetness index estimates soil moisture content, computed as a function of local slope angle and the neighboring maximum values of drainage area [45].

Soil fertility was assessed from an average of 70 soil samples collected in each 6,25-ha plot between 2004 and 2006. Firstly, we sampled along catenas with topography as stratifying variables; and secondly in area locally under-sampled (S1a in S1 Text). Each soil sample was collected in the topsoil at 10−20 cm depth, using an hand-auger of 10 cm diameter. For each sample, thirteen chemical parameters were analyzed related to three soil characteristics: (i) organic matter with total C and N contents, and C/N ratio; (ii) soil nutrients with soil saturation of exchange bases (CEC, S, BS, Na, Mg, K, Ca) and available phosphorus content (P); and (iii) soil acidity with content of H and Al exchangeable. To reduce the number of variables we used a Principal Component Analysis (S1b in S1 Text) to select five soil fertility variables (Table 1) considered to be representative of the three soil characteristics and to be important for the floristic composition [46–48] directly or indirectly by reflecting physical constraints [49]. Thus we selected total C content, both chemical compounds related to soil acidity (H and Al exchangeable) because Al has also a toxic effect on plants [50], the base-cation saturation ratio (BS) and the available P content. The latter two chemical variables represent nutrients available for plants that have heterogeneous and limited distribution at a local spatial scale, and so are considered as indicator of fertility in tropical soils [51]. We created raster maps of soil properties for each of the permanent sample plots using unbiased linear interpolation by kriging (S1c-f in S1 Text).

### Association test

To test pairwise association of spatially structured raster maps, we used a recently developed method that controls for bias due to spatial autocorrelation [29]. They proposed a Monte Carlo test of Pearson’s product-moment correlation coefficient between two maps (r_obs_) based on replicated pairs simulated by an image synthesis technique that preserves the autocorrelation function of each original map. We used 999 simulations to determine the p-value of each observed correlation between two raster maps. In our analysis, the p-value is considered as a continuous variable measuring “the strength of evidence against the null hypothesis” [52] of an absence of correlation between two raster maps. As p-values are bounded to 0.001 with 999 Monte Carlo simulations, we fitted a normal distribution to the simulated correlations (r_sim_) from which we approximated the expected p-value that |r_obs_| > |r_sim_|. Maps were standardized by default to 32 x 32 cells, which corresponds to a resolution of 7.8 m per cell, achieved via data smoothing.

We used the image synthesis method in two ways. First, we examined correlations between pairs of environmental variables to understand relations between variables and to select key representative variables for subsequent analyses. This analysis was realized on plot 15 only, because it is the only plot which has relatively equal representation of contrasting topographic habitats (i.e., plateaus and bottomslopes). We then tested for species-habitat associations by examining correlations among the selected environmental variables and raster maps of species abundances for the 94 focal tree species. Density raster maps for each species were created by convoluting occurrence count of species per 7.8 m cell with a two-dimensional Gaussian with a standard deviation of 1.15 cells. A symmetric extension was applied before the convolution to prevent edge effect. Independent random synthetic images were generated for each plot, but we studied the correlation for each species-habitat association for all plots combined together.

### Habitat preferences and growth strategies

We further examined if species with similar habitat preferences also shared functional strategies. We estimated growth strategy using three traits, the maximum diameter (90^th^ percentile) across the permanent plot inventories; the maximum growth rate (90^th^ percentile) in these inventories between 1991 and 2013 [53]; and the wood density measured as wood specific gravity [34]. We conducted two analyses. First, we examined all 94 taxa, testing correlations (Spearman test) between our index of habitat preference: the p-value of the association test [52] and each of the three functional traits. Second, we used phylogenetically-independent contrasts of congeneric species pairs that exhibited contrasting habitat preferences. We used Wilcoxon signed-rank tests to determine if shifts in habitat preferences were consistently accompanied by shifts of functional trait values in the same direction.

### Data analyses

Most analyses were realized with the free software R [54] and more specifically with following packages: *raster* [55], *rgdal* [56] and *maptools* [57] for cartogarphic tools; *RGeostats* [58] for kriging;. SAGA GIS (System for Automated Geoscientific Analysis Geographic Information Systems) free terrain analysis software was used to create hydrological maps from DTM [43]. MATLAB software [59] was used to perform maps' association tests.

## Results

### Environmental variables of soil heterogeneity

**Fig 2:**
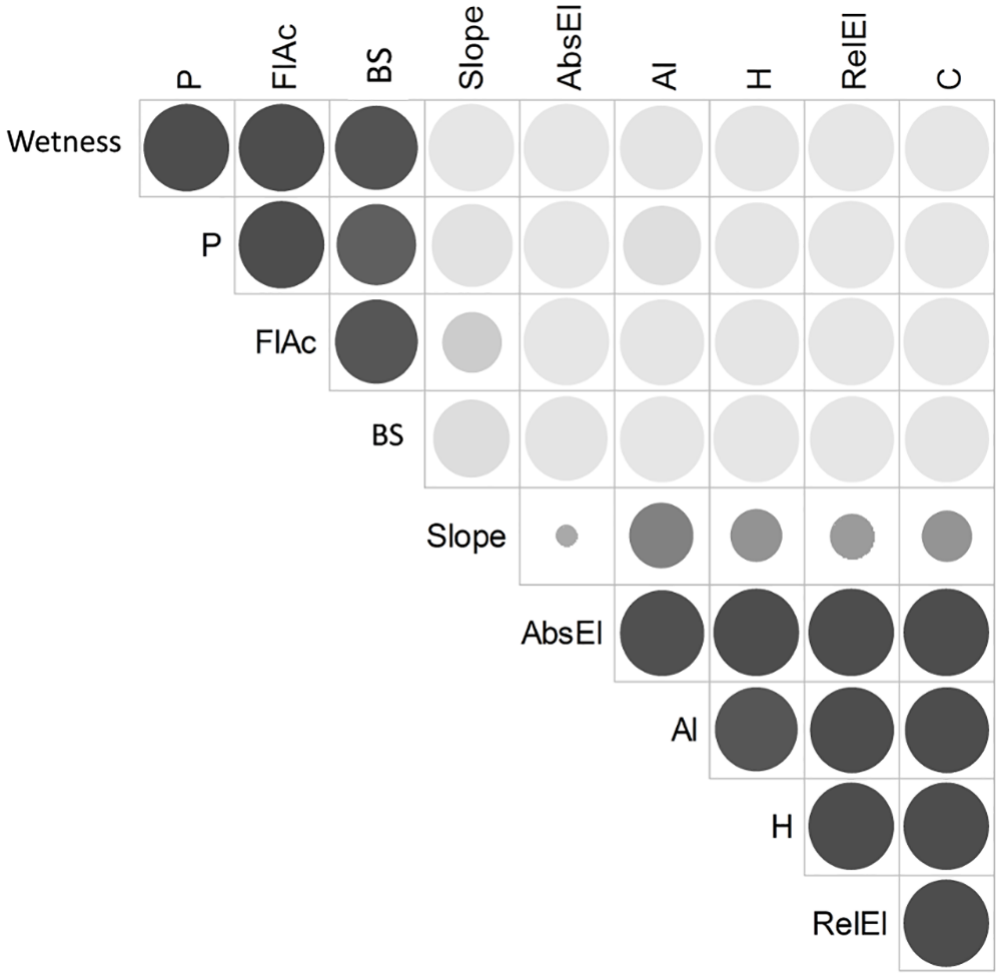
Correlation plot between environmental variables. The color of the circles indicates the sign of the correlation (highly positive in black to highly negative in light grey), while the size of circles indicates the level of statistical significance of the association test (the lower the p-value, the bigger the circle). Environmental variables are: wetness index (Wetness), available phosphorus content (P), flow accumulation (FlAc), base-cation saturation ratio (BS), local slope angle (Slope), absolute elevation (AbsEl), Al exchangeable (Al), H exchangeable (H), relative elevation (RelEl) and total C content (C).

Based on the results of environmental map association tests (Fig 2), two variables were retained to describe soil heterogeneity: relative elevation and local slope angle. We observed two groups of variables (Wetness, P, FlAc, BS vs. AbsEl, Al, H, RelEl, C), which were positively correlated within each group and negatively correlated between groups. Local slope angle variable was negatively correlated with the first group and not correlated with the second group. We could thus contrast low elevation and hydromorphic areas corresponding to seasonally inundated bottomlands, against high elevation and non-hydromorphic areas corresponding to plateaus. Soil fertility variables were distributed accordingly, with fertile soils (high content of P and BS) on seasonally inundated bottomlands, and acidic soils rich in organic matter (high content of H, Al and C) on plateaus. To avoid redundancy in subsequent analyses, we chose only one variable to represent the two correlated groups. We selected relative elevation because it was easily derived from the LIDAR DTM and more consistently interpretable in terms of soil hydrological regime than absolute elevation. We also retained the local slope angle as a variable providing complementary information on soil features.

### Species distribution along environmental gradients

**Fig 3:**
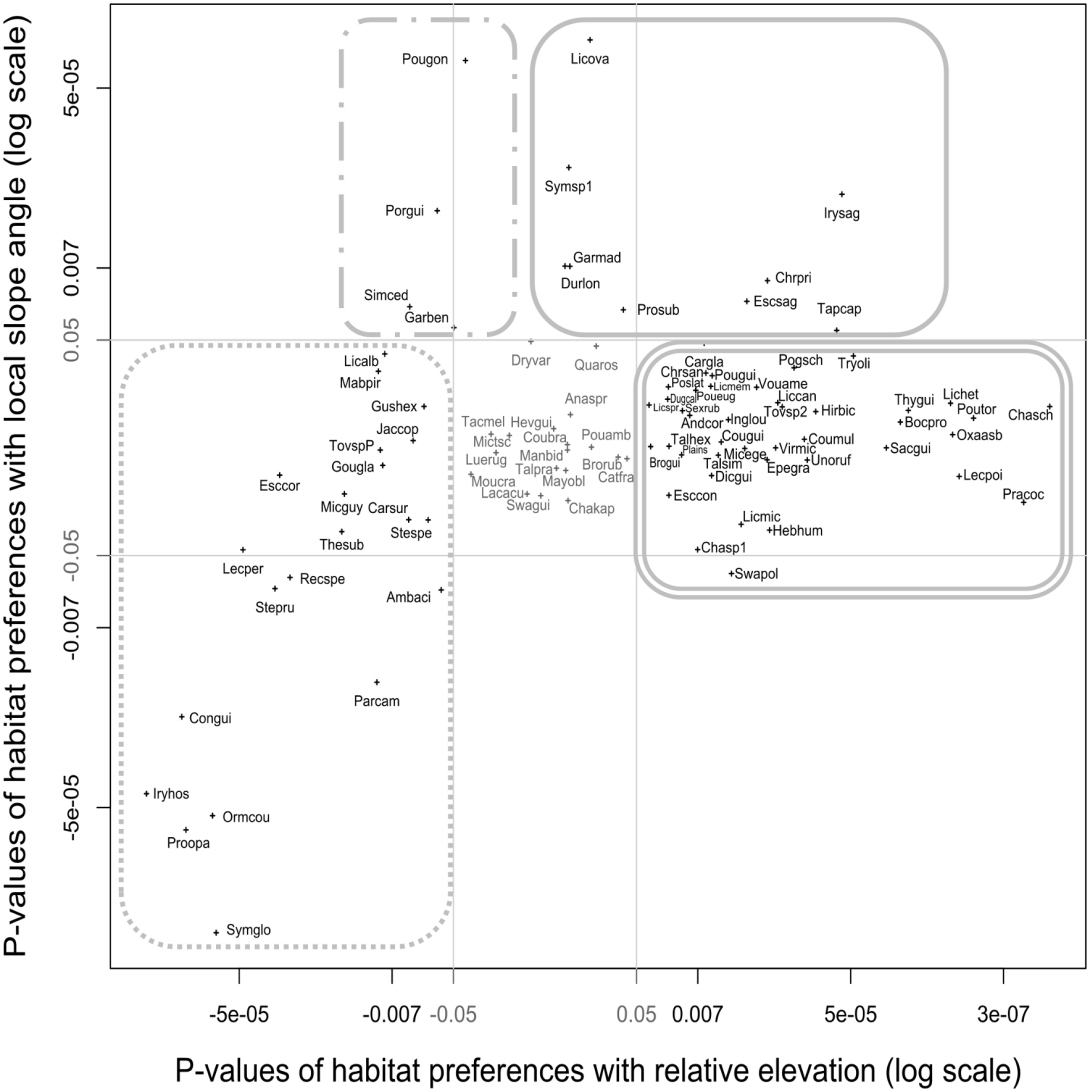
Species-habitat associations with respect to relative elevation from the nearest stream and local slope angle. Each axis represents the strength of the species-habitat association measured as p-values of Monte Carlo tests of species density and environmental raster maps correlations based on an image synthesis technique (see Methods). P-values are indicated in log scale with a sign indicating whether the correlation with the environmental variable is positive or negative. Grey lines correspond to absolute p-value of 0.05. Species’ codes are given in S1 Table. Encircling lines delineate four different species groups (see Text).

Habitat preferences explain the spatial structuration of a significant number of species (Table 2), with 74.5% of species significantly varying in abundance with relative elevation and 27.7% of species significantly varying in abundance with local slope angle. Contrasting habitat preferences with seasonally inundated bottomlands (24.5% of species) and plateaus (48.9% of species) were detected across elevation gradient for the majority of species. Significant species preference (13.8%) or avoidance (10.6%) for slopes was also detected. Four groups of species were thus identified in ordinations of p-values of the association test (Fig 3 and S2 Table): (i) species associated with flat plateaus (Fig 3, in double line), (ii) species associated with top of slopes (Fig 3, in simple line), (iii) species associated with bottomslopes (Fig 3, in dashed line), (iv) species associated with flat seasonally inundated bottomlands near the main streams (Fig 3, in dot line).

**Table 2:** Frequency of species-habitat associations.

	Significant correlations		
	Total	Positive	Negative
**RelEl**	73.4%	48.9%	24.5%
**Slope**	24.4%	13.8%	10.6%

Frequency of significant (p ≤ 0.05) species-habitat associations with relative elevation above the nearest stream (RelEl) and local slope angle (Slope). P-values come from a Monte Carlo test of raster maps correlations based on an image synthesis technique (see Methods).

We observed a high frequency of genera exhibited contrasting habitat preferences for congeneric species (70% of the 14 genera with congeneric species that have a significant association test), including genera *Lecythis*, *Eschweilera*, *Micropholis*, *Tovomita* and *Licania* with respect to plateaus and seasonally inundated bottomlands; genera *Iryanthera*, *Symphonia*, *Eschweilera*, *Protium* and *Licania* with respect to top of slope and seasonally inundated bottomlands; genus *Garcinia* with respect to top of slope and bottom slope; genus Pouteria with respect to plateaus and bottom slope (see S2 Table). Only some genera (30% of the 14 genera) were specific of particular habitats, such as *Sterculia* on seasonally inundated bottomlands, *Talisia* and *Chaetocarpus* on plateaus, *Chrysophyllum* on top of slope.

### Habitat preferences and functional growth strategies

**Fig 4:**
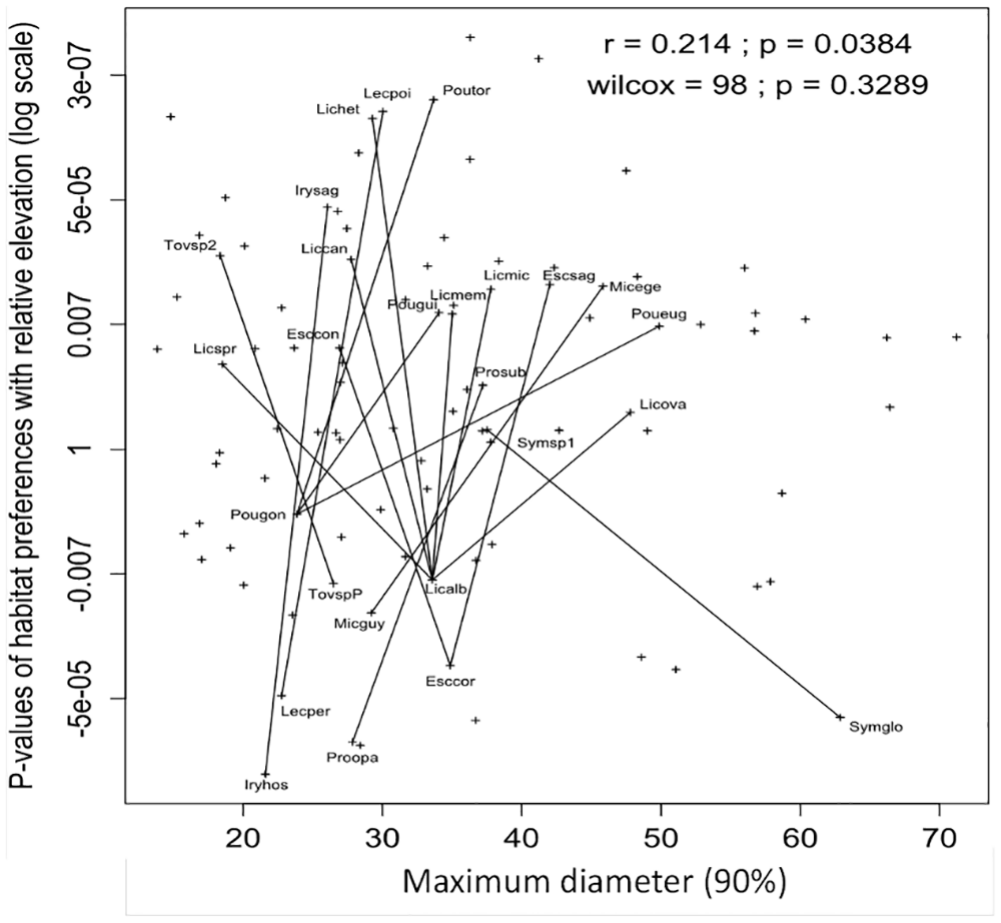
Species-habitat associations and functional strategies. X-axis represents maximum diameter of each species. Y-axis represents the strength of the species-habitat association measured as P-values of Monte Carlo tests of species density and relative elevation from the nearest stream raster maps correlations based on an image synthesis technic (see Methods). P-values are indicated in log scale with a sign indicating whether the correlation with the environmental variable is positive or negative. Pearson's *r* indicates the correlation between species values on x- and y-axis, while Wilcoxon's W is performed between tree diameter maximum of congeneric species pairs with contrasting habitat preferences, linked by dark lines. Species’ codes are given in S1 Table.

We observed a positive relationship between our index of species-habitat associations for relative elevation and the maximum diameter (r = 0.21, p < 0.05; Fig 4); that is, species associated with plateaus tended to be of larger stature (maximum DBH) than species associated with seasonally inundated bottomlands. Nevertheless, this observation was not consistent within tree lineages comprising contrasting habitat specialists (W = 98, p = 0.33). In particular, smaller statured species of *Eschweilera*, *Symphonia* and *Tovomita* are associated with plateaus rather than seasonally inundated bottomlands (Fig 4). No trend of growth strategies was observed among species preferences with local slope angle.

## Discussion

### Species-habitat associations

We found that topographic variables are strong proxies for soil hydrology, which correlates with a combination of physico-chemical properties. This is consistent with several studies that have used topography as a proxy for soil hydrological functioning [20,60]. Soil hydrological properties result from weathering sequences along topographical catena that have been described at local scales in French Guiana [39,42]. This includes (i) a thick and vertically well-drained initial ferralitic cover with clayey and humic horizons; (ii) apparition of a compact weathering horizon on slope, as a result of mechanical and chemical erosion of the initial cover, which modifies the soil drainage from deep and vertical to superficial and lateral; (iii) apparition of an hydromorphic system at the bottom of slopes where the upper horizons are more or less permanently saturated by a slow lateral flow coming from the slope, and in connection with the main drainage axes. Contrary to [14] who suggested that soil fertility gradients provide information on habitat preferences that is complementary to topographic data, in our study site, soil fertility varied predictably across topographic gradients. Indeed, leaching of soils from well-drained plateaus to seasonally inundated bottomlands explained the decrease in clay content, the increase in nutrient content (BS) and acidity decrease (H and Al exchangeable). Clay content, being positively correlated with total C content [37], explains higher total C content on plateaus than on seasonally inundated bottomlands. We observed a higher concentration of available phosphorus on seasonally inundated bottomlands than plateaus because phosphorus is released during the reduction of iron (Fe^3+^ to Fe^2+^) under hydromorphic conditions which are anaerobic [61]. These results are concordant with Amazon-wide analyses showing strong correlations between soil fertility and soil physical properties, with well-drained soils often being highly weathered and nutrient depleted [62]. Because both (i) our results were consistent with previous works realized on similar sites in soil structure, and (ii) these topographical variables being easily derived from LIDAR DTM, we propose that LIDAR-derived variables may efficiently permit a stratification of soil without analysis of soil samples in sites similar in soil structure to those studied here.

We found a pervasive pattern of species-habitat associations with two topographic variables at Paracou, with almost three-quarters of the 94 tree species studied associated with relative elevation from the nearest stream and/or local slope angle. Other studies on broader scales [63,64] found evidences for strong effects of soil characteristics, such as topography and geomorphology, on the distribution of species. However, our study addressed on finer spatial scale (with more precise measure of soil properties and taking into consideration replicates that account for spatial aggregation) is complementary to these previous studies because combination of both local and short-term mechanisms as well as regional and long-term processes were crucial for a complete understanding of soil in structuring these communities [65]. Many authors suggest that topography is one of the major determinants of species distributions and community patterns at small scales, typically 1–50 ha [10,15,18,66,67]. In our study site, we observed that a combination of these two topographical variables represents a good proxy for soil type, resulting from weathering sequences along topographical catena. Thus species-habitat associations with these topographic variables correspond to preferences for soil hydrological and physico-chemical properties and not topographic characteristics *per se*. Tolerance of species to prolonged water saturation is the main factor that explains the species distribution. Indeed, 69 of 94 species revealed habitat preferences related to relative elevation, which represents a gradient of decreasing tolerance to prolonged water saturation [42]. Only 23 species revealed habitat preferences related to local slope angle. Four main groups of habitat specialists can be identified across the two complementary topographic variables: (i) species associated with flat plateaus, which are tolerant to acid, humic, clayey and well-drained soil (Fig 3, in double line); (ii) species associated with top of slopes, which are tolerant to soil under mechanical and chemical erosion due to superficial and lateral soil drainage (Fig 3, in simple line); (iii) species associated with bottomslopes, which are tolerant to hydromorphic soil with accumulation of nutrients leached from the top of slope (Fig 3, in dashed line); (iv) species associated with flat seasonally inundated bottomlands, which are tolerant to fertile and constraining hydromorphic soil (Fig 3, in dot line).

### Ecological and evolutionary trends in species-habitat associations

We found some general ecological patterns linking functional strategies with species-habitat associations. We observed two guilds of species along topographic gradients with species preferring plateaus that tend to have larger maximum size than species on seasonally inundated bottomlands. Topographic gradients at Paracou have also been linked to forest dynamics, with higher gap frequency and therefore light availability on seasonally inundated bottomlands [35], which may limit maximum stature in these habitats. Nevertheless, we found no general pattern linking functional proxies of growth rates with topography as predicted, for example, by [34].

We also observed that habitat preferences have evolved repeatedly in different tree lineages, such that there is no niche conservatism for distributions of species across soil gradients in this region. Instead, we observed that closely-related species were frequently associated with different habitats. The frequency of evolutionary diversification events related to contrasting habitat preferences in this and other studies [20,30] suggests the importance of environmental filtering in tropical tree community assembly. Moreover, it cautions against substituting data on habitat preferences for understudied congeneric taxa.

We expected some coordination between our results of functional strategies and congeneric contrasts, with habitat specialists from different genera sharing functional strategies [30]. However, we found that phylogenetically-independent contrasts of functional strategies were not consistent among congeneric species pairs with contrasting habitat preferences; that is, species on seasonally inundated bottomlands did not always have smaller stature than congeneric plateau species (Fig 4). A possible explanation would be that we still lack data on ecophysiological traits such as cavitation risk that might discriminate different growth strategies depending on hydrological conditions [31]. Moreover, it’s important to consider that species-habitat associations may also be due to processes other than strict environmental filtering, including dispersal limitation [7,18,68], forest dynamics [15,35], or interspecific relationships such as competition or shared natural enemies [69]. Indeed these processes influence species distribution and could be confound with environmental effects. In future analyses it could be interesting to decouple the relative contribution of each of these processes. Moreover, a comprehensive integration of evolutionary and ecological approaches would expand from the local spatial scale studied here to a larger biogeographic area that would include entire lineages so that true sister species contrasts might be examined, with appropriate resolution of phylogenetic hypotheses. However, such an approach is only feasible for single smaller clades [5,70].

In conclusion, we demonstrated that a combination of two topographical variables easily derived from LIDAR DTM (relative elevation and local slope angle) represents a good proxy for soil type in this tropical forest, resulting from weathering processes along topographical gradients. With this proxy, we demonstrated pervasive habitat preferences of species for soil hydrological and physico-chemical properties. Our results suggest that species-habitat associations could be link to some general functional strategies and could be consistent with diversification events. These ecological and evolutionary trends could have contributed to hyperdiverse regional species pools in the region, in addition to the maintenance of local diversity.

## Supporting Information

S1 File Maps of topography. Rasters maps of topography are stored in a multi-layers raster that contains: (i) a raster layer of absolute elevation corresponding to the Digital terrain model (DTM) derived from LIDAR (Light Detection And Ranging) data (in m); (ii) a raster layer of relative elevation corresponding to the relative altitude above the nearest stream (in m); (iii) a raster layer of local slope angle corresponding to the estimation of slope angle deviation from the horizontal plane (in degrees).
(TIF)

S2 File Maps of soil hydrology. Rasters maps of soil hydrology are stored in a multi-layers raster that contains: (i) a layer of wetness index corresponding to the estimation of soil moisture; (ii) a layer of flow accumulation corresponding to the estimation of surface runoff.
(TIF)

S3 File Maps of soil fertility. Rasters maps of soil fertility are stored in a multi-layers raster that contains: (i) a layer of total C content (in g.kg^-1^); (ii) a layer of available P content (in mg.kg^-1^); (iii) a layer of Al exchangeable (in cmolc.kg^-1^); (iv) a layer of H exchangeable (in cmolc.kg^-1^); (v) a layer of exchange bases soil-saturation (BS in %).
(TIF)

S1 Text Conception of soil fertility maps by kriging. (PDF)

S1 Table Abundance of species. Column “Labels” corresponds to the first three letters both genus and specie. Columns “P1”, “P6”, “P11”, “P13”, “P14” and “P15” corresponds respectively to abundance in plot 1, 6, 11, 13, 14 and 15. Column “Total” corresponds to total abundance for the six plots. Column “Ind_ha” corresponds to a number of individual per ha.
(PDF)

S2 Table Results of association test between densities of species and the two topographic variables. Column “Labels” correspond to the first three letters both genus and species. Column “p_RelEl” corresponds to the expected p-value that |r_obs_| > |r_sim_| that we approximated by fitting a normal distribution to the simulated correlations (r_sim_) of association tests between species density and relative elevation. Column “r_RelEl” corresponds to the coefficient of correlation of association test between species density and relative elevation. Column “p_Slope” correspond to the expected p-value that |r_obs_| > |r_sim_| that we approximated by fitting a normal distribution to the simulated correlations (r_sim_) of association tests between species density and local slope angle. Column “r_Slope” corresponds to the coefficient of correlation of association tests between species density and local slope angle.
(PDF)
