# Supplementary material for: Pervasive Local-Scale Tree-Soil Habitat Association in a Tropical Forest Community
Source: PLoS One. 2015 Nov 4;10(11):e0141488. doi: 10.1371/journal.pone.0141488 (PMC4633048; doi:10.1371/journal.pone.0141488)
Supplement: S1 Text — (PDF) [file pone.0141488.s004.pdf]

Supporting Information, text S1.

Conception of soil fertility maps by kriging

a. Localization of soil samples in each plot

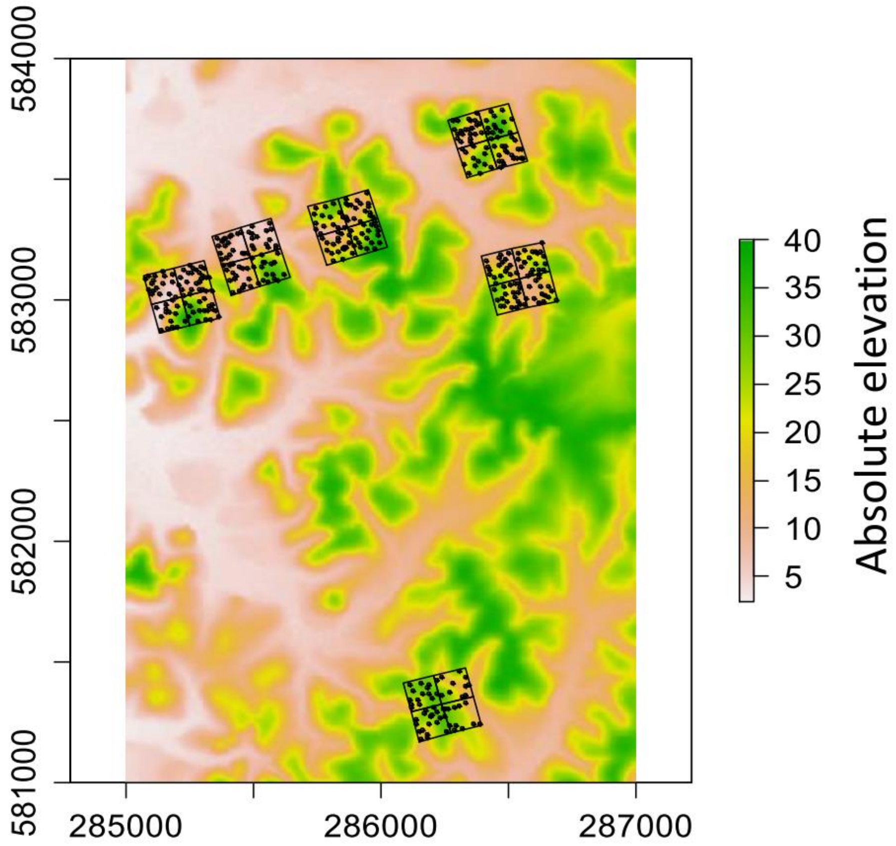

Soil fertility was assessed from an average of 70 soil samples collected in each 6.25-ha plot between 2004 and 2006. Firstly, we sampled along catenas with topography as stratifying variables; and secondly in area locally under-sampled. Each soil sample was collected in the topsoil at 10-20 cm depth, using an hand-auger of 10 cm diameter. For each sample, thirteen chemical parameters were analyzed related to three soil characteristics: (i) organic matter with total C and N contents, and ratio C/N; (ii) soil nutrient with soil saturation of exchange bases (CEC, S, BS, Na, Mg, K, Ca) and available phosphorus content (P); and (iii) soil acidity with content of H and Al exchangeable.

b. Principal Component Analysis of values of the thirteen chemical parameters analyzed for each soil samples

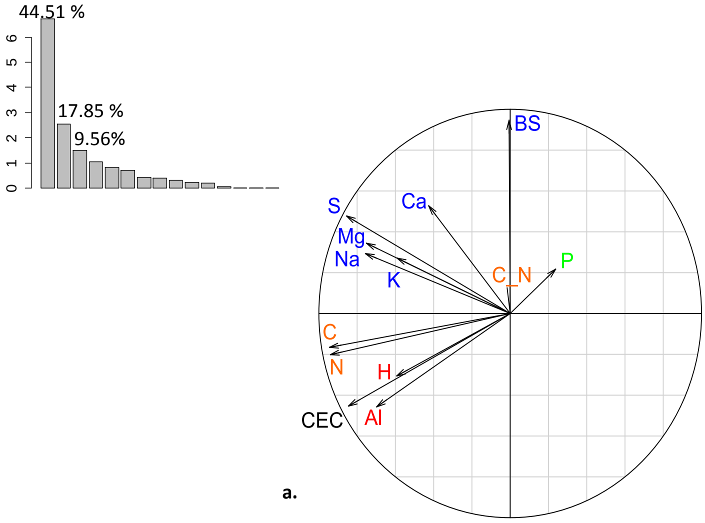

71.9% of the variance of soil fertility values is explained by the first three axes of the Principal Component Analysis (PCA). a) ordination on PC 1 and 2 and b) ordination on PC 1 and 3. The first axis reflects organic matter of soil (in orange with C and N corresponding respectively to total C and N content) and the exchangeable complex (with Ca, Mg, Na, K, Al, H, S corresponding to soil saturation of exchange bases, CEC to cation-exchange capacity). The second axis distinguishes the nature of exchangeable cations between saturation of exchange bases (in blue) and soil acidity (in red). The third axis reflects soil nutrients with available P content (in green). For subsequent analyses we selected five soil fertility variables to reduce the number of variables while being representative of the three soil characteristics, choosing the parameters considered important for the floristic composition and according to this PCA. Thus we selected total C content that represents the organic matter, both chemical compounds related to soil acidity (H and Al exchangeable) because Al has also a toxic role on the floristic composition (Scholl et al. 2004), the base-cation saturation ratio (BS), and the available P content.

c. Steps of kriging

The first step consists to estimate spatial autocorrelation of a variable, using an experimental variogram that defines semi-variance of points at different distance classes. The second step consists to establish a model that fits the experimental variogram. The third step is the kriging of spatial data compared to the prior model construct. For this study, distances of experimental variogram were defined from 0 to 176 m (by default, half the length of the diagonal) by 20 m distance classes. Experimental variograms are anisotropic, that is spatial autocorrelation are observed in four directions (0, 45, 90, 135°). Outlier values of samples are not retained for the experimental variogram. To define outliers' values for each variable, distributions of variables values are observed and the threshold of values retained was defined by testing models with different thresholds by cross-validation. Cross-validation was realized by comparing original values to model values, and data with cross-validation near to 1 were retained. Ordinary kriging, was applied on a regular grid with nodes every 5 m. For each of the five retained soil fertility variables, we derived raster maps illustrating kriging estimates.

d. Selection of the five chemical compounds' range values for kriging by the index of cross-validation

Compounds	Range of values without selection	Range of values with selection	Index of cross-validation for values without selection	Index of cross-validation with selection
**C**	[0 ; 4,5[	**[0 ; 4[**	5,0930	**0,9582**
**P**	[0 ; 25]	**[0 ; 10[**	6,2832	**0,9419**
**H**	**[0 ; 1,2[**	[0 ; 0,8[	**1,0238**	1,1358
**Al**	[0 ; 3[	**[0 ; 2,2[**	1,0572	**1,0127**
**BS**	[0 ; 85[	**[0 ; 20[**	1,1700	**1,0900**

Outlier values of samples are not retained for the experimental variogram. To define outliers' values for each variable, distributions of variables values are observed and the range of values retained was defined by testing models from data with different range by cross-validation. Cross-validation was realized by comparing original values to model values, and range of values with index of cross-validation near to 1 were retained (in bold).

e. Model characteristics used to krige the five chemical compounds

Model characteristics		C	P	H	Al	BS
**Nugget effect**	Sill	0,048	1,601		0,012	4,477
**Cubic model**	Sill	0,087	0,411			
	Anisotropy	1 ; 0	1 ; 2,483			
	Ranges	0,002 - 60,248	151,656 - 61,073			
	Angle	35,939 degrees	132,6 degrees			
**Gaussian model**	Sill			0,019		
	Anisotropy			1 ; 0,854		
	Ranges			0,026 - 0,031		
	Angle			169,532 degrees		
**Exponential model**	Sill			7,201	0,041	
	Anisotropy			1 ; 0,402	1 ; 698300,873	
	Ranges			129619,034 - 322315,237	360,438 - 0,001	
	Angle			30,383 degrees	-30,736 degrees	
**Spherical model**	Sill	0,231	1,382		0,09	5,838
	Anisotropy	1 ; 0,782	1 ; 0,622		1 ; 0,642	1 ; 0,743
	Ranges	118,8 - 151,919	152,339 - 244,849		167,248 - 260,695	266,483 - 358,746
	Angle	9,517 degrees	-39,443 degrees		-49,505 degrees	-110,931 degrees
**Total sill**		0,366	3,394	7,221	0,143	10,315

f. Data, models and results of kriging for each five chemical parameters

We created maps of soil properties for each of the permanent sample plots using an unbiased method of linear interpolation: kriging methods. We used an R package named RGeostats.

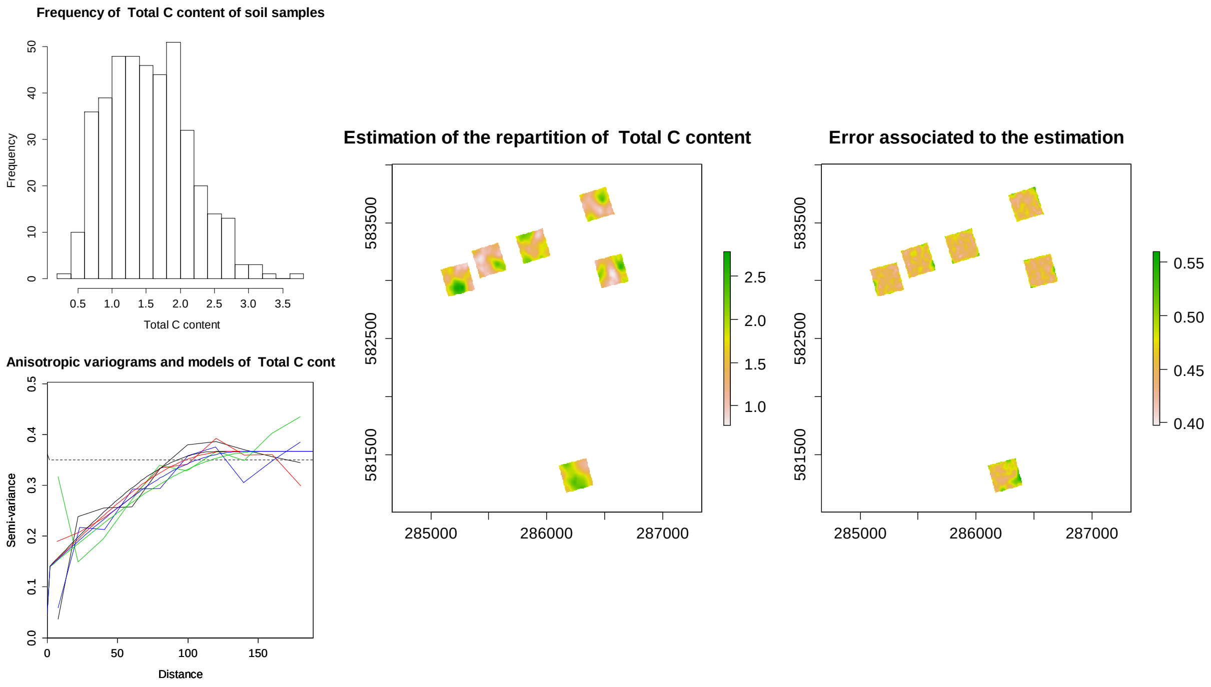

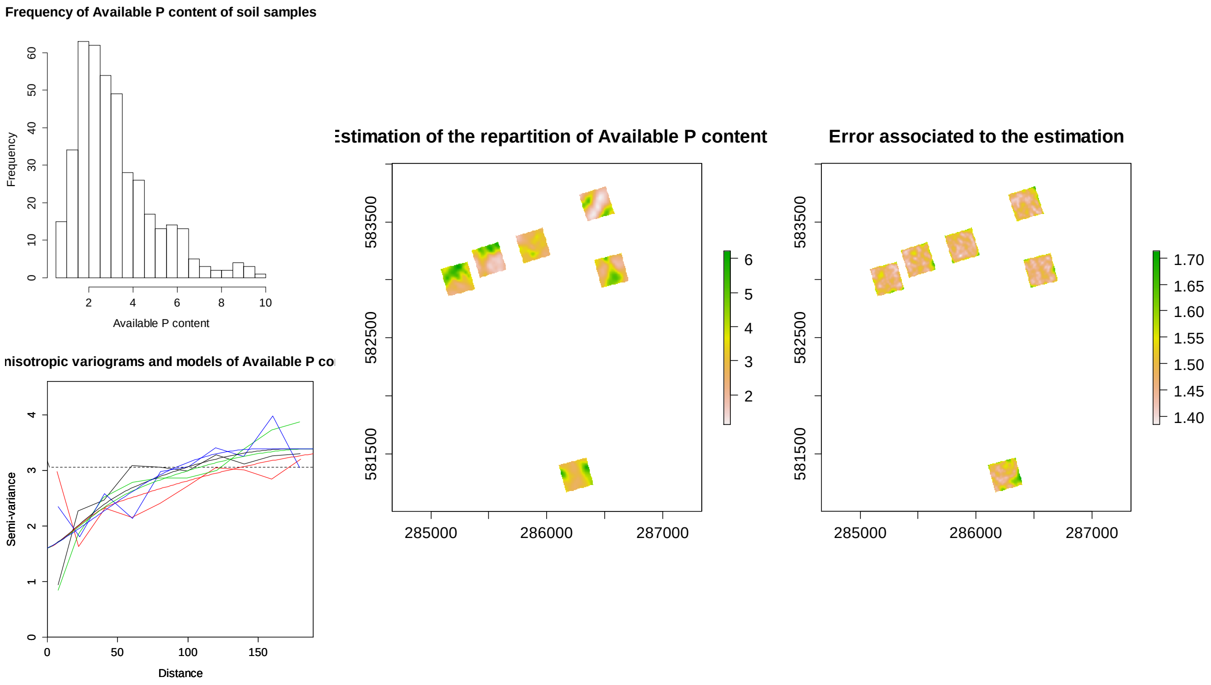

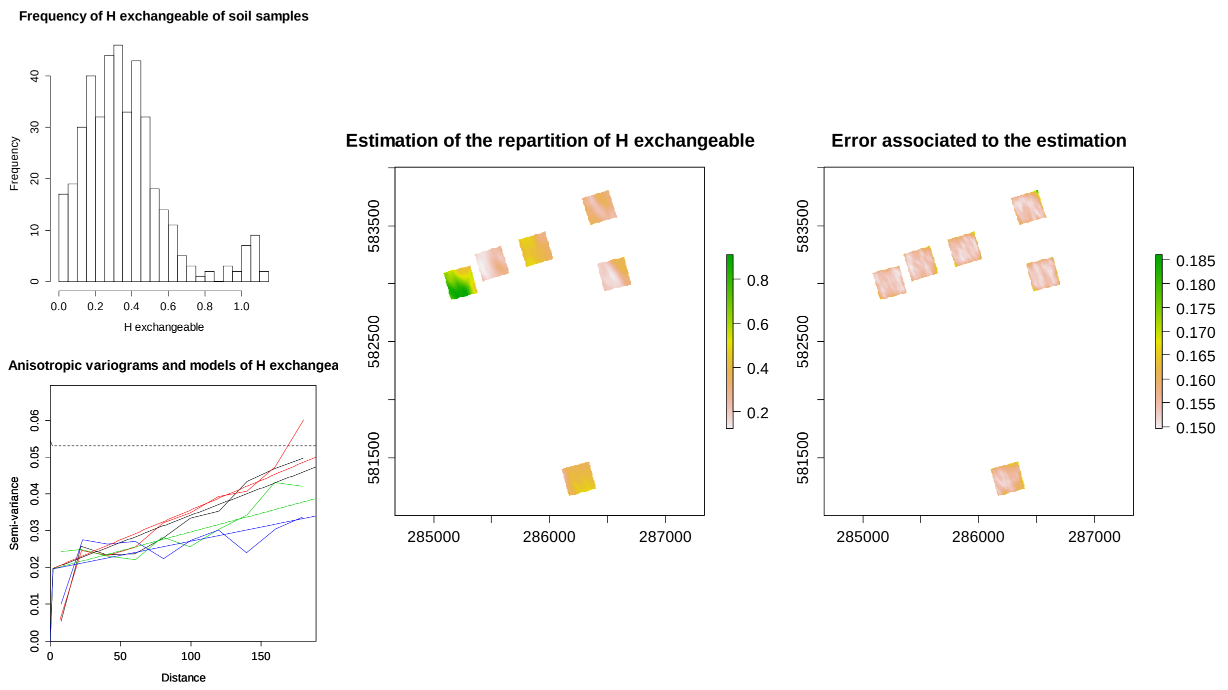
Frequency of H exchangeable of soil samples
Frequency
0
10
20
30
40
0.0
0.2
0.4
0.6
0.8
1.0
H exchangeable
Anisotropic variograms and models of H exchangea
Semi-variance
0.00
0.01
0.02
0.03
0.04
0.05
0.06
0
50
100
150
Distance
Estimation of the repartition of H exchangeable
583500
582500
581500
285000
286000
287000
0.8
0.6
0.4
0.2
Error associated to the estimation
583500
582500
581500
285000
286000
287000
0.185
0.180
0.175
0.170
0.165
0.160
0.155
0.150

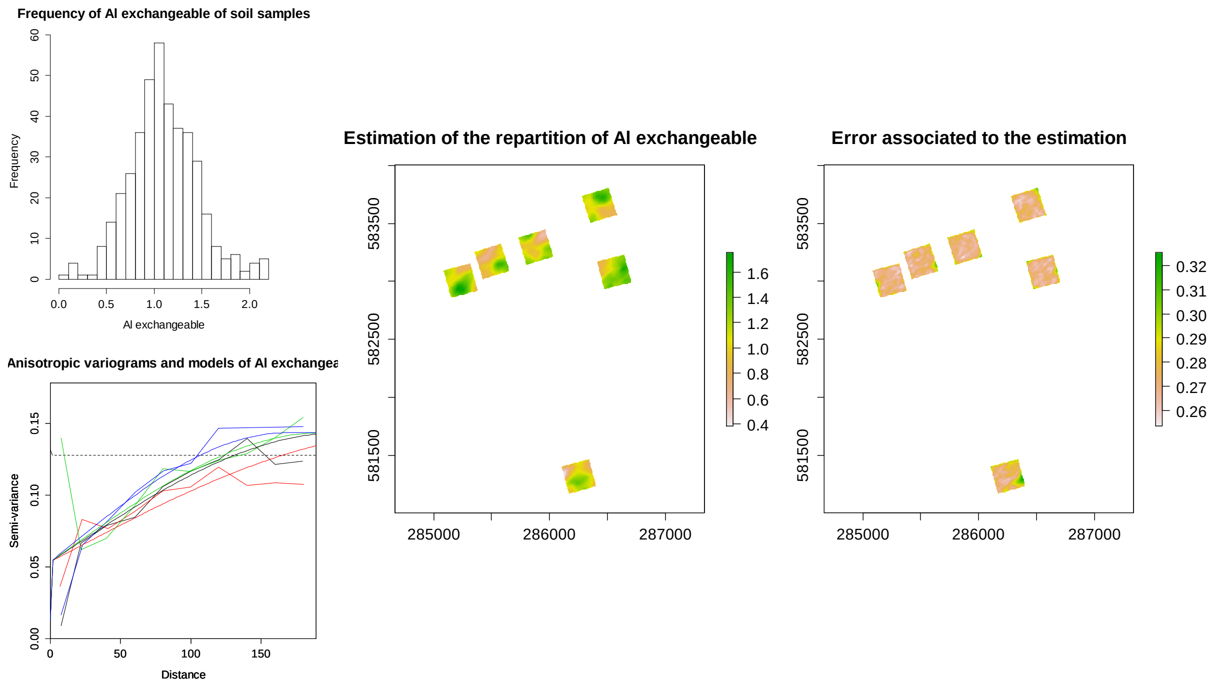
Frequency of Al exchangeable of soil samples
Frequency
0
10
20
30
40
50
60
0.0
0.5
1.0
1.5
2.0
Al exchangeable
Anisotropic variograms and models of Al exchangea
Semi-variance
0.00
0.05
0.10
0.15
0
50
100
150
Distance
Estimation of the repartition of Al exchangeable
583500
582500
581500
285000
286000
287000
1.6
1.4
1.2
1.0
0.8
0.6
0.4
Error associated to the estimation
583500
582500
581500
285000
286000
287000
0.32
0.31
0.30
0.29
0.28
0.27
0.26

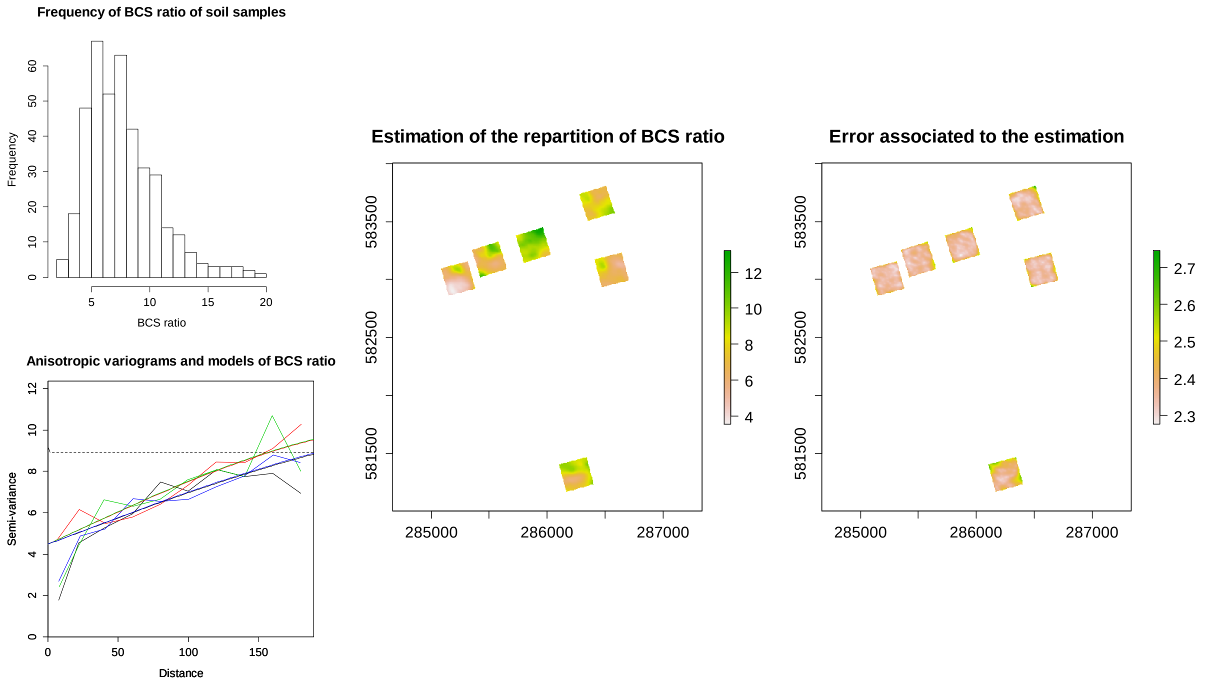

Different steps of kriging is described for the five chemical parameters (total C content, available P content, H exchangeable, Al exchangeable and base-cation saturation ration). Description of the organization of one panel:

- on top left of the panel, values' distribution of the chemical compound considered for kriging
- on bottom left, estimation of spatial autocorrelation of chemical compound's values, using an experimental variogram that defines semi-variance of points at different distance classes. An automatic model is then established by fitting the experimental variogram. For this study, distances of experimental variogram were defined from 0 to 176 m (by default, half the length of the diagonal) by 20 m distance classes. Experimental variograms are anisotropic, that is spatial autocorrelation are observed in four directions (0, 45, 90, 135°)
- in the center, map of kriging estimation of repartition of the chemical compound consider, compared to the prior model construct. Ordinary kriging, was applied on a regular grid with nodes every 5 m.
- on right, map of kriging error associated to estimation
