# Supplementary material for: Pervasive Local-Scale Tree-Soil Habitat Association in a Tropical Forest Community
Source: PLoS One. 2015 Nov 4;10(11):e0141488. doi: 10.1371/journal.pone.0141488 (PMC4633048; doi:10.1371/journal.pone.0141488)
Supplement: S1 Table — Column “Labels” corresponds to the first three letters both genus and specie. Columns “P1”, “P6”, “P11”, “P13”, “P14” and “P15” corresponds respectively to abundance in plot 1, 6, 11, 13, 14 and 15. Column “Total” corresponds to total abundance for the six plots. Column “Ind_ha” corresponds to a number of individual per ha. (PDF) [file pone.0141488.s005.pdf]

Supporting Information, table S1.

Abundance of species

Species	Labels	P1	P6	P11	P13	P14	P15	Total	Ind_ha
Ambelania_acida	Ambaci	3	4	12	13	9	18	59	1,57
Anacardium_spruceanum	Anaspr	10	2	8	4	9	9	42	1,12
Andira_coriacea	Andcor	6	7	6	3	7	10	39	1,04
Bocoa_prouacensis	Bocpro	78	47	91	100	86	80	482	12,85
Brosimum_guianense	Brogui	1	16	11	8	13	16	65	1,73
Brosimum_rubescens	Brorub	6	10	7	12	5	12	52	1,39
Carapa_surinamensis	Carsur	34	36	47	31	28	32	208	5,55
Caryocar_glabrum	Cargla	9	9	12	4	11	7	52	1,39
Catostemma_fragrans	Catfra	15	16	41	24	23	34	153	4,08
Chaetocarpus_schomburgkianus	Chasch	13	28	40	12	36	32	161	4,29
Chaetocarpus_sp.1	Chasp1	7	8	21	6	6	5	53	1,41
Chaunochiton_kappleri	Chakap	8	6	13	13	2	7	49	1,31
Chrysophyllum_prieurii	Chrpri	18	15	32	9	20	18	112	2,99
Chrysophyllum_sanguinolentum	Chrsan	6	8	17	7	29	16	83	2,21
Conceveiba_guianensis	Congui	17	22	7	18	1	13	78	2,08
Couepia_bracteosa	Coubra	8	12	14	6	10	3	53	1,41
Couepia_guianensis	Cougui	6	3	13	7	3	10	42	1,12
Couratari_multiflora	Coumul	28	33	67	29	27	23	207	5,52
Dicorynia_guianensis	Dicgui	33	42	56	35	22	37	225	6
Drypetes_variabilis	Dryvar	6	5	11	16	17	14	69	1,84
Duguetia_calycina	Dugcal	6	4	16	5	19	17	67	1,79
Duroia_longiflora	Durlon	8	8	8	4	15	7	50	1,33
Eperua_grandiflora	Epegra	22	66	162	65	41	33	389	10,37
Eschweilera_congestiflora	Esccon	9	15	29	15	6	22	96	2,56
Eschweilera_coriacea	Esccor	62	36	9	23	40	8	178	4,75
Eschweilera_sagotiana	Escsag	221	151	276	255	266	308	1477	39,39
Garcinia_benthamiana	Garben	14	1	4	25	5	26	75	2
Garcinia_madruno	Garmad	1	2	4	6	8	21	42	1,12
Goupia_glabra	Gougla	17	18	18	13	22	11	99	2,64
Gustavia_hexapetala	Gushex	43	38	48	34	37	72	272	7,25
Hebepetalum_humiriifolium	Hebhum	8	11	36	16	9	23	103	2,75
Hevea_guianensis	Hevgui	9	24	16	15	9	9	82	2,19
Hirtella_bicornis	Hirbic	21	8	23	12	5	13	82	2,19
Inga_loubryana	Inglou	12	8	23	8	16	12	79	2,11
Iryanthera_hostmannii	Iryhos	59	21	17	87	19	24	227	6,05
Iryanthera_sagotiana	Irysag	49	27	24	32	50	30	212	5,65
Jacaranda_copaia	Jaccop	13	15	15	11	7	9	70	1,87
Lacmellea_aculeata	Lacacu	4	4	8	10	7	9	42	1,12
Lecythis_persistens	Lecper	290	202	258	243	259	222	1474	39,31

Lecythis_poiteaui	Lecpoi	14	11	30	13	18	22	108	2,88
Licania_alba	Licalb	210	112	201	192	178	131	1024	27,31
Licania_canescens	Liccan	42	47	3	31	50	27	200	5,33
Licania_heteromorpha	Lichet	76	26	117	61	93	150	523	13,95
Licania_membranacea	Licmem	75	30	80	71	38	37	331	8,83
Licania_micrantha	Licmic	23	9	30	47	20	30	159	4,24
Licania_ovalifolia	Licova	13	11	12	12	14	21	83	2,21
Licania_sprucei	Licspr	10	11	16	17	18	31	103	2,75
Lueheopsis_rugosa	Luerug	9	1	2	10	8	10	40	1,07
Mabea_piriri	Mabpir	11	11	1	17	13	7	60	1,6
Manilkara_bidentata	Manbid	4	2	19	9	7	7	48	1,28
Maytenus_oblongata	Mayobl	14	10	14	12	3	18	71	1,89
Miconia_tschudyoides	Mictsc	3	7	17	1	17	4	49	1,31
Micropholis_egensis	Micege	4	9	6	5	9	16	49	1,31
Micropholis_guyanensis	Micguy	10	4	7	18	12	11	62	1,65
Mouriri_crassifolia	Moucra	12	15	36	25	32	18	138	3,68
Ormosia_coutinhoi	Ormcou	15	6	6	25	9	6	67	1,79
Oxandra_asbeckii	Oxaasb	75	116	165	87	97	125	665	17,73
Parinari_campestris	Parcam	8	4	16	16	5	4	53	1,41
Platonia_insignis	Plains	9	1	14	5	4	15	48	1,28
Pogonophora_schomburgkiana	Pogsch	70	120	173	78	67	135	643	17,15
Poraqueiba_guianensis	Porgui	19	10	7	9	27	15	87	2,32
Posoqueria_latifolia	Poslat	9	6	45	7	18	8	93	2,48
Pouteria_ambelaniifolia	Pouamb	5	7	11	4	8	11	46	1,23
Pouteria_eugeniifolia	Poueug	7	7	10	7	5	23	59	1,57
Pouteria_gongrijpii	Pougon	26	7	9	2	14	7	65	1,73
Pouteria_guianensis	Pougui	21	3	11	6	7	20	68	1,81
Pouteria_torta	Poutor	10	3	19	3	12	15	62	1,65
Pradosia_cochlearia	Pracoc	20	55	101	23	58	29	286	7,63
Protium_opacum	Proopa	36	20	28	41	21	7	153	4,08
Protium_subserratum	Prosub	8	7	3	12	9	9	48	1,28
Qualea_rosea	Quaros	76	1	83	23	5	1	189	5,04
Recordoxylon_speciosum	Recspe	27	30	31	44	34	42	208	5,55
Sacoglottis_guianensis	Sacgui	12	7	9	7	5	9	49	1,31
Sextonia_rubra	Sexrub	20	10	10	9	14	17	80	2,13
Simaba_cedron	Simced	53	27	40	34	33	31	218	5,81
Sterculia_pruriens	Stepru	14	20	17	26	19	23	119	3,17
Sterculia_speciosa	Stespe	8	14	9	2	4	7	44	1,17
Swartzia_guianensis	Swagui	9	8	16	18	3	15	69	1,84
Swartzia_polyphylla	Swapol	6	11	11	16	13	16	73	1,95
Symphonia_globulifera	Symglo	24	5	6	27	10	11	83	2,21
Symphonia_sp.1	Symsp1	58	75	47	54	83	80	397	10,59
Tachigali_melinonii	Tacmel	6	9	6	3	11	4	39	1,04
Talisia_hexaphylla	Talhex	10	10	13	11	5	16	65	1,73
Talisia_praealta	Talpra	6	5	8	8	4	8	39	1,04
Talisia_simaboides	Talsim	5	3	8	7	4	12	39	1,04

Tapura_capitulifera	Tapcap	19	63	2	30	45	134	293	7,81
Theobroma_subincanum	Thesub	24	17	22	21	13	21	118	3,15
Thyrsodium_guianense	Thygui	15	14	7	1	8	22	67	1,79
Tovomita_sp.2_DS	Tovsp2	16	19	24	13	25	26	123	3,28
Tovomita_sp.P4	TovspP	10	6	8	29	9	18	80	2,13
Trymatococcus_oligandrus	Tryoli	8	3	9	6	17	22	65	1,73
Unonopsis_rufescens	Unoruf	3	2	2	6	6	41	60	1,6
Virola_michelii	Virmic	10	7	24	4	4	2	51	1,36
Vouacapoua_americana	Vouame	74	89	10	70	99	22	364	9,71
