# Supplementary material for: Pervasive Local-Scale Tree-Soil Habitat Association in a Tropical Forest Community
Source: PLoS One. 2015 Nov 4;10(11):e0141488. doi: 10.1371/journal.pone.0141488 (PMC4633048; doi:10.1371/journal.pone.0141488)
Supplement: S2 Table — Column “Labels” correspond to the first three letters both genus and species. Column “p_RelEl” corresponds to the expected p-value that |robs| > |rsim| that we approximated by fitting a normal distribution to the simulated correlations (rsim) of association tests between species density and relative elevation. Column “r_RelEl” corresponds to the coefficient of correlation of association test between species density and relative elevation. Column “p_Slope” correspond to the expected p-value that |robs| > |rsim| that we approximated by fitting a normal distribution to the simulated correlations (rsim) of association tests between species density and local slope angle. Column “r_Slope” corresponds to the coefficient of correlation of association tests between species density and local slope angle. (PDF) [file pone.0141488.s006.pdf]

Supporting Information,table S2.

Results of association test between densities of species and the two topographic variables

Species	Labels	p_RelEl	r_RelEl	p_Slope	r_Slope
Ambelania_acida	Ambaci	0,03354971	-0,119	0,01920946	-0,106
Anacardium_spruceanum	Anaspr	0,43472756	0,04	0,39757632	0,033
Andira_coriacea	Andcor	0,00877892	0,13	0,40638289	0,037
Bocoa_prouacensis	Bocpro	8,96E-06	0,44	0,48912587	0,04
Brosimum_guianense	Brogui	0,03152088	0,11	0,96626955	0,002
Brosimum_rubescens	Brorub	0,0914553	0,093	0,77167682	-0,012
Carapa_surinamensis	Carsur	0,01158679	-0,163	0,13550011	-0,078
Caryocar_glabrum	Cargla	0,00547958	0,144	0,05429658	0,085
Catostemma_fragrans	Catfra	0,06837917	0,088	0,73840299	-0,013
Chaetocarpus_schomburgkianus	Chasch	6,77E-08	0,269	0,31766774	0,042
Chaetocarpus_sp.1	Chasp1	0,00677648	0,121	0,05899362	-0,088
Chaunochiton_kappleri	Chakap	0,47024556	0,04	0,2308654	-0,046
Chrysophyllum_prieurii	Chrpri	0,00069148	0,217	0,00958749	0,119
Chrysophyllum_sanguinolentum	Chrsan	0,00517802	0,117	0,12510815	0,059
Conceveiba_guianensis	Congui	6,94E-06	-0,281	0,00056596	-0,172
Couepia_bracteosa	Coubra	0,48031777	0,031	0,91548479	0,004
Couepia_guianensis	Cougui	0,00314129	0,171	0,85067103	0,008
Couratari_multiflora	Coumul	0,00020687	0,28	0,78810269	0,015
Dicorynia_guianensis	Dicgui	0,00430229	0,186	0,46213293	-0,034
Drypetes_variabilis	Dryvar	0,6313073	-0,024	0,05199616	0,084
Duguetia_calycina	Dugcal	0,01807205	0,136	0,25893583	0,045
Duroia_longiflora	Durlon	0,51912705	0,036	0,00640547	0,105
Eperua_grandiflora	Epegra	0,00069843	0,299	0,71658354	-0,021
Eschweilera_congestiflora	Esccon	0,01733978	0,172	0,26737999	-0,051
Eschweilera_coriacea	Esccor	0,00017032	-0,335	0,46748106	-0,041
Eschweilera_sagotiana	Escsag	0,00135763	0,289	0,01706629	0,11
Garcinia_benthamiana	Garben	0,05095387	-0,11	0,03540467	0,092
Garcinia_madruno	Garmad	0,44251074	0,048	0,00643913	0,116
Goupia_glabra	Gougla	0,00495984	-0,183	0,61301907	-0,021
Gustavia_hexapetala	Gushex	0,01914165	-0,176	0,31684376	0,048
Hebepetalum_humiriifolium	Hebhum	0,00064386	0,261	0,10149292	-0,08
Hevea_guianensis	Hevgui	0,75291411	0,022	0,5884682	0,028
Hirtella_bicornis	Hirbic	0,00014247	0,25	0,36573654	0,046
Inga_loubryana	Inglou	0,00248498	0,171	0,4576276	0,034
Iryanthera_hostmannii	Iryhos	2,19E-06	-0,488	6,67E-05	-0,253
Iryanthera_sagotiana	Irysag	6,05E-05	0,322	0,00086904	0,18
Jacaranda_copaia	Jaccop	0,01340936	-0,116	0,81901097	0,011

Lacmellea_aculeata	Lacacu	0,55732136	-0,031	0,27715617	-0,042
Lecythis_persistens	Lecper	5,13E-05	-0,338	0,05869396	-0,1
Lecythis_poiteaui	Lecpoi	1,31E-06	0,292	0,45161556	-0,031
Licania_alba	Licalb	0,00530456	-0,266	0,0735474	0,086
Licania_canescens	Liccan	0,00049295	0,257	0,28544216	0,057
Licania_heteromorpha	Lichet	1,73E-06	0,515	0,28976366	0,06
Licania_membranacea	Licmem	0,00441574	0,209	0,18126638	0,061
Licania_micrantha	Licmic	0,00163324	0,207	0,11912733	-0,067
Licania_ovalifolia	Licova	0,22881598	0,062	1,19E-05	0,185
Licania_sprucei	Licspr	0,03322833	0,13	0,30442734	0,042
Lueheopsis_rugosa	Luerug	0,20195162	-0,064	0,87279191	-0,007
Mabea_piriri	Mabpir	0,00429984	-0,158	0,11917843	0,064
Manilkara_bidentata	Manbid	0,47916138	0,045	0,9393756	-0,003
Maytenus_oblongata	Mayobl	0,50621303	0,038	0,53235722	-0,026
Miconia_tschudyoides	Mictsc	0,31193584	-0,046	0,7101149	0,016
Micropholis_egensis	Micege	0,00145267	0,184	0,98172562	-0,001
Micropholis_guyanensis	Micguy	0,0014169	-0,188	0,27726497	-0,049
Mouriri_crassifolia	Moucra	0,08887299	-0,108	0,4789373	-0,032
Ormosia_coutinhoi	Ormcou	1,89E-05	-0,367	3,62E-05	-0,242
Oxandra_asbeckii	Oxaasb	1,62E-06	0,502	0,69311742	0,022
Parinari_campestris	Parcam	0,00410154	-0,153	0,00147719	-0,139
Platonia_insignis	Plains	0,01144307	0,132	0,82156276	-0,009
Pogonophora_schomburgkiana	Pogsch	0,00029014	0,319	0,10804447	0,08
Poraqueiba_guianensis	Porgui	0,02946927	-0,124	0,00137229	0,133
Posoqueria_latifolia	Poslat	0,01767519	0,12	0,18374049	0,06
Pouteria_ambelaniifolia	Pouamb	0,218782	0,074	0,98202057	0,001
Pouteria_eugeniifolia	Poueug	0,00720859	0,146	0,20240723	0,049
Pouteria_gongrijpii	Pougon	0,07424272	-0,105	2,11E-05	0,19
Pouteria_guianensis	Pougui	0,00421745	0,181	0,13474165	0,07
Pouteria_torta	Poutor	8,18E-07	0,27	0,43858357	0,031
Pradosia_cochlearia	Pracoc	1,59E-07	0,398	0,21965079	-0,049
Protium_opacum	Proopa	7,96E-06	-0,334	2,44E-05	-0,221
Protium_subserratum	Prosub	0,07713514	0,1	0,02159089	0,098
Qualea_rosea	Quaros	0,18688714	0,086	0,05918779	0,103
Recordoxylon_speciosum	Recspe	0,00023927	-0,264	0,02721644	-0,114
Sacoglottis_guianensis	Sacgui	1,41E-05	0,315	1	0
Sextonia_rubra	Sexrub	0,01116122	0,141	0,3565277	0,042
Simaba_cedron	Simced	0,01198782	-0,156	0,01983298	0,112
Sterculia_pruriens	Stepru	0,00014644	-0,236	0,01997028	-0,102
Sterculia_speciosa	Stespe	0,02188887	-0,104	0,13469232	-0,056
Swartzia_guianensis	Swagui	0,87179912	-0,008	0,26414311	-0,05
Swartzia_polyphylla	Swapol	0,00224049	0,207	0,03031893	-0,093
Symphonia_globulifera	Symglo	2,14E-05	-0,324	1,40E-06	-0,263
Symphonia_sp.1	Symsp1	0,45776074	0,045	0,00041374	0,164
Tachigali_melinonii	Tacmel	0,17133144	-0,072	0,684146	0,017
Talisia_hexaphylla	Talhex	0,01729869	0,11	0,95924715	0,002

Talisia_praealta	Talpra	0,68700778	0,016	0,56741669	-0,02
Talisia_simaboides	Talsim	0,00345956	0,167	0,81495953	-0,008
Tapura_capitulifera	Tapcap	7,18E-05	0,367	0,03826675	0,107
Theobroma_subincanum	Thesub	0,00129979	-0,242	0,09717172	-0,086
Thyrsodium_guianense	Thygui	6,90E-06	0,325	0,35333464	0,039
Tovomita_sp.2_DS	Tovsp2	0,00042626	0,307	0,32017011	0,05
Tovomita_sp.P4	TovspP	0,00457289	-0,147	0,9386134	-0,003
Trymatococcus_oligandrus	Tryoli	4,16E-05	0,262	0,07775375	0,087
Unonopsis_rufescens	Unoruf	0,00018807	0,261	0,71190163	-0,018
Virola_michelii	Virmic	0,00052932	0,201	1	0
Vouacapoua_americana	Vouame	0,00098553	0,288	0,18591931	0,076
